# 

**Published:** 1849-09

**Authors:** 


### NOTICE TO READERS AND CORRESPONDENTS.

Thanks to our numerous contributors, we are able to present a number of our Journal filled with original matter entire. We shall be able to select from the accumulated file, the most interesting and ably written articles for each successive number hereafter.

The general interest which the Profession has taken in the subject of Cholera has induced us to give an unusual amount of attention to the subject in the present and preceding numbers of the Journal, and to extend the size of each, which will be made right in quantity in the course of the volume. We would thank our friends throughout the country where it has prevailed, to send us accounts of the appearance and progress of Cholera as bearing upon the subject of its communicable nature, whether favoring or disproving it.

We learn from one of our agents that Dr. J. M. Logan, Sec'y of the Æsculapean Society, has sent by him original papers for the Journal---by Dr. S. Thompson, of Albion, Ill., and Dr. J. M. Logan, of Palestine, Ill., which have not yet come to hand.

We have received from the publishers, Barth & Rogers' Manual of Auscultation: For sale by S. C. Griggs & Co. Quain & Sharpeys' Anatomy, for sale by Jos. Keen & Bro.

Original communications have been received from Drs. Ritchie, Mathews and Jones, and an exceedingly unmannerly epistle from a Dr. Craig, of Indiana.

We have received from Prof. Horner, the Circular of St. Joseph's Hospital, of which we will give a fuller notice hereafter.

---

### RECEIPTS FOR THIS JOURNAL FOR JULY AND AUGUST, 1849.

### IOWA.

| | |
|---|---|
| Drs. W. Reinhart, | $2 00 |
| J. P. Crothers, | 2 00 |
| S. L. Craig, | 1 00 |
| H. Howey, | 2 00 |
| H. Rousseau, | 3 00 |
| F. Knowles, | 2 00 |
| M. F. Collins, | 4 00 |
| C. H. Ober, | 2 00 |
| E. R. Ford, | 2 00 |
| C. W. Cowles, | 2 00 |
| Miller & Norris, | 2 00 |
| J. N. Anderson, | 1 00 |

### WISCONSIN.

| | |
|---|---|
| Drs. W. H. Walker, | $2 50 |
| N. C. Rowley, | 2 00 |
| A. H. Bowie, | 3 00 |
| B. F. Mills, | 3 00 |
| Earll & Thornhill, | 2 00 |
| Wm. Lewis, | 4 00 |
| David Walker, | 2 00 |
| W. W. Blackman, | 3 00 |
| J. Parker, | 2 00 |
| J. T. Buckley, | 1 00 |
| R. T. Goodwin, | 2 00 |
| S. D. Wallace, | 2 00 |
| J. H. & A. B. Wright, | 2 00 |
| R. Earll, | 2 00 |

### ILLINOIS.

| | |
|---|---|
| Drs. W. W. Welch. | $2 00 |
| D. K. Town, | 6 00 |
| D. D. Wait, | 2 00 |
| E. Sanford, | 2 00 |
| W. L. Hutchinson, | 2 00 |
| A. W. Benton, | 2 00 |
| W. S. Gray, | 1 00 |
| J. H. Brown, | 2 00 |
| G. J. Huey, | 2 00 |
| P. A. Allaire, | 6 00 |
| W. H. Winter, | 4 00 |
| J. Davidson, | 2 00 |
| H. Cutler, | 2 00 |
| W. C. Snyder, | 2 00 |
| Drs. D. E. Ellis, | 3 00 |
| L. F. Avery, | 2 00 |
| J. S. Bullock, | 4 00 |
| W. W. Hopkins, | 4 00 |
| D. W. Boyd, | 1 00 |
| J. Burritt, | 2 00 |
| M. A. Smith, | 5 00 |
| S. R. Haven, | 2 00 |
| H. H. Hinman, | 1 00 |
| E. Winslow, | 2 00 |
| R. B. Landon, | 1 00 |
| T. A. Hill, | 2 00 |
| W. O. Chamberlain, | 2 00 |
| E. S. Morey, | 2 00 |
| J. Grover, | 2 00 |
| W. A. Norman, | 4 00 |

### OHIO.

| | |
|---|---|
| Dr. N. Strong. | 2 00 |

### INDIANA.

| | |
|---|---|
| Drs. J. S. Collins, | 2 00 |
| W. Townsend, | 2 00 |
| Jos. Ogden, | 2 00 |
| P. Gibbons, | 2 00 |
| E. Van Buskirk, | 3 00 |
| S. Grimes, | 2 00 |
| S. Loftin, | 2 00 |
| W. C. Farrington, | 6 00 |
| Jno. F. Parks, | 2 00 |
| S. W. Ritchey, | 2 00 |
| E. H. Sutton, | 3 00 |
| W. R. Nofsinger, | 4 00 |
| T. Bullard, | 2 00 |
| H. Courtney, | 2 00 |

### MICHIGAN.

| | |
|---|---|
| Drs. J. W. Hall, | 2 00 |
| Chas. Shepherd, | 2 00 |
| L. Tompkins, | 2 00 |
| H. Sheldon, | 5 00 |
| Campbell & Cox, | 2 00 |
| Goodrich, | 2 00 |
| J. Babcock, | 2 00 |
| R. S. Hawley, | 4 00 |
| F. A. Bailey, | 4 00 |

